# Biphasic Thyroid-Like Low-Grade Nasopharyngeal Papillary Adenocarcinoma with a Prominent Spindle Cell Component: A Case Report

**DOI:** 10.3390/diagnostics10050323

**Published:** 2020-05-19

**Authors:** Sang Hwa Lee, Hyunjin Kim, Min Ju Kim, Byungwha Kim, Hyun-Soo Kim

**Affiliations:** 1 Pathology Center, Seegene Medical Foundation, Seoul 04805, Korea; lsh@mf.seegene.com; 2 Department of Pathology and Translational Genomics, Samsung Medical Center, Sungkyunkwan University School of Medicine, Seoul 06351, Korea; h2407.kim@samsung.com; 3 Department of Pathology, Soonchunhyang University College of Medicine, Bucheon Hospital, Bucheon 14584, Korea; mjkim80127@naver.com; 4 Luke Ear, Nose, and Throat Clinic, Donghae 25769, Korea; kimbw4@hanmail.net

**Keywords:** nasopharynx, thyroid-like low-grade nasopharyngeal papillary adenocarcinoma, prominent spindle cell component, immunohistochemistry, thyroid transcription factor-1

## Abstract

Thyroid-like low-grade nasopharyngeal papillary adenocarcinoma (TLLG-NPPA) is a distinctly rare malignancy of the nasopharynx. Morphologically and immunophenotypically, TLLG-NPPA resembles papillary thyroid carcinoma (PTC) and is characterized by a papillary architecture with PTC-like nuclear features and thyroid transcription factor-1 expression. Recently, some cases of TLLG-NPPA with a spindle cell component have been reported. In this study, we report a very interesting case of biphasic TLLG-NPPA that was predominantly composed of spindle cells, with comprehensive analyses of its clinical, pathological, and immunophenotypical features. A 50-year-old woman presented with a sensation of a foreign body in the nasopharynx. Nasopharyngoscopy and computed tomography demonstrated a pedunculated mass arising from the nasopharyngeal roof. Based on the clinical impression of a nasopharyngeal tumor, an excisional biopsy was performed. At low-power magnification, the nasopharyngeal mass consisted of papillary tumor tissue, the growth pattern and architecture of which resembled those of PTC. The papillae were complex and packed tightly with fibrovascular cores. At high-power magnification, each papillary structure was lined with a pseudostratified cuboidal-to-columnar epithelium. The tumor cell nuclei frequently showed a ground-glass appearance, intranuclear grooves, pseudoinclusions, and membrane thickening and irregularity, resembling the characteristic nuclear morphology of PTC. These histological features were compatible with TLLG-NPPA. Intriguingly, in between the papillary components were spindle cells that appeared very similar to the glandular epithelial cells that imperceptibly merged with the papillary component. This spindle cell component comprised two-thirds of the entire tumor volume. The nuclear morphology of the spindle cell component was similar to that of the papillary component. On immunostaining, both the papillary and spindle cell components were diffusely and strongly positive for thyroid transcription factor-1, cytokeratin 7, cytokeratin 19, vimentin, and Hector Battifora mesothelial-1. In contrast, the tumor cells tested negative for p63, p40, smooth muscle actin, S-100, cytokeratin 5/6, thyroglobulin, BRAF V600E, and Epstein–Barr virus-encoded small RNAs. Only two cases of biphasic TLLG-NPPA exhibiting a prominent spindle cell component had been reported previously in the English literature. When the pathologist receives a primary nasopharyngeal mass with the aforementioned histological features, particularly biopsy specimens with predominant spindle cells, biphasic TLLG-NPPA should be considered in the differential diagnosis. By describing its detailed clinicopathological characteristics, we anticipate that this report will expand the existing knowledge on the spindle cell component associated with TLLG-NPPA.

## 1. Introduction

Primary malignant tumors of the nasopharynx can arise from different origins, including epithelial, mesenchymal, hematological, and neurogenic. Among them, epithelial tumors are classified into nasopharyngeal carcinoma, nasopharyngeal papillary adenocarcinoma, and salivary gland-type carcinoma, according to the 2017 World Health Organization Classification of Head and Neck Tumors [1]. While nasopharyngeal carcinoma is the most common type of malignancy of the nasopharynx, nasopharyngeal papillary adenocarcinoma is distinctly uncommon and comprises approximately 0.5% of all nasopharyngeal malignancies [2,3,4]. Primary nasopharyngeal adenocarcinoma arises from two sources (submucosal seromucinous glands and surface mucosal epithelia), each of which exhibits distinct clinicopathological characteristics. The former presents in an older age group (mean, 50 years) and is more aggressive than its surface mucosal counterpart. It encompasses various histological types analogous to carcinomas of the salivary glands, including polymorphous low-grade adenocarcinoma, mucoepidermoid adenocarcinoma, and adenoid cystic carcinoma [5,6]. On the other hand, adenocarcinomas arising from the surface mucosal epithelium are usually low grade and characterized by a papillary configuration, young age of onset (mean, 37 years), and an excellent prognosis. For these tumors, complete surgical excision is often curative.

In 1988, Wenig et al. [6] first referred to a primary nasopharyngeal adenocarcinoma arising from the surface mucosal epithelium as a low-grade nasopharyngeal papillary adenocarcinoma, because this entity was found to have a biological potential of low-grade malignancy. Since then, case reports have rarely been published. Histologically, low-grade nasopharyngeal papillary adenocarcinoma is characterized by papillary fronts and crowded glandular structures lined with cuboidal-to-columnar epithelium resembling a papillary thyroid carcinoma (PTC) [5,6]. In 2005, Carrizo and Luna [7] first documented the expression of thyroid transcription factor-1 in tumor cell nuclei in two patients with low-grade nasopharyngeal papillary adenocarcinoma. Based on these immunostaining results in conjunction with the histological features, the authors coined the term thyroid-like low-grade nasopharyngeal papillary adenocarcinoma (TLLG-NPPA). Recently, Petersson et al. [2] reported the presence of a spindle cell component in a patient with TLLG-NPPA. Only six cases of TLLG-NPPA exhibiting spindle cells have been reported to date [2,3,8,9,10]. Here, we present another case of biphasic TLLG-NPPA that contained a prominent spindle cell component in a 50-year-old woman. We anticipate that this report will expand our current knowledge on this rare entity and particularly of the spindle cell component associated with TLLG-NPPA, by describing the clinicopathological characteristics and immunophenotype, and by reviewing the previously published literature.

## 2. Case Report

### 2.1. Clinical Presentation

**Figure 1:**
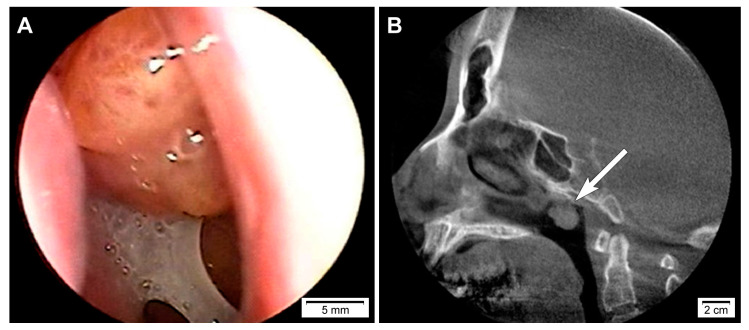
Imaging findings of biphasic thyroid-like low-grade nasopharyngeal papillary adenocarcinoma. (**A**) Nasopharyngoscopy and (**B**) computed tomography scan revealed a pedunculated mass arising from the roof of the nasopharynx (long white arrow).

This study (2020-05-104) was reviewed and approved by the Institutional Review Board of Samsung Medical Center (Seoul, Republic of Korea). A 50-year-old woman presented with a sensation of a foreign body in the nasopharynx which she had never experienced before. Physical examination revealed no obvious abnormality. Nasopharyngoscopy (Figure 1A) and computed tomography scan (Figure 1B) revealed a pedunculated mass arising from the roof of the nasopharynx. Thyroid ultrasonography did not reveal any remarkable findings. Based on the clinical impression of a nasopharyngeal tumor, an excisional biopsy was performed. Written informed consent could not be obtained for the publication of this case report and its accompanying images because the patient was referred to another tertiary hospital immediately after the diagnosis.

### 2.2. Pathological Features

The biopsied specimens were fixed in 10% neutral-buffered formalin and embedded in paraffin blocks. From each formalin-fixed, paraffin-embedded block, 4-μm sections were cut and stained with hematoxylin and eosin. The most representative hematoxylin and eosin-stained slide was chosen for immunostaining.

**Figure 2:**
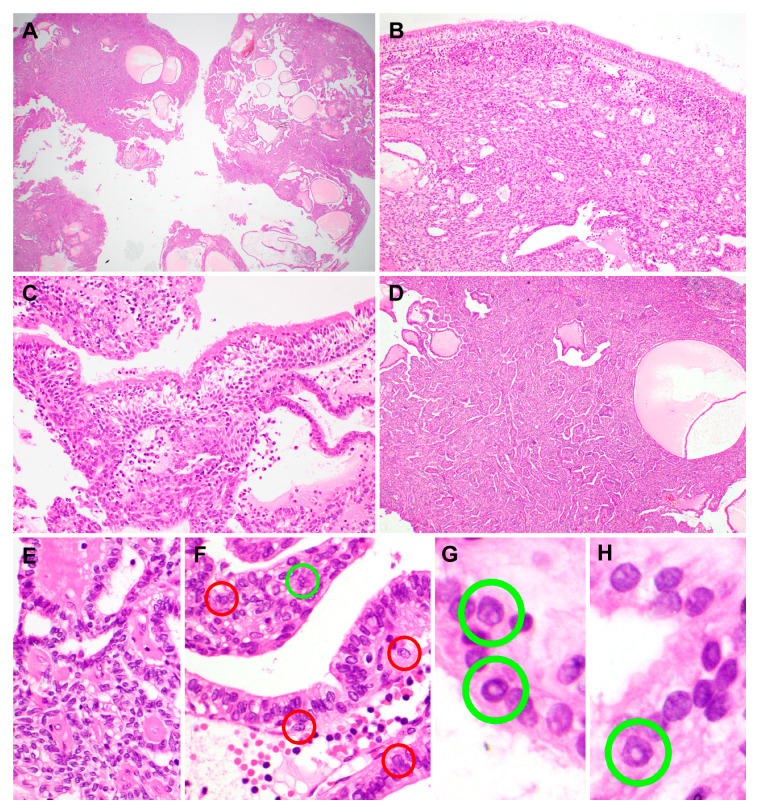
Histopathological features of the papillary component of biphasic thyroid-like low-grade nasopharyngeal papillary adenocarcinoma. (**A**) On a scan view, the tumor was solid and cystic, without necrosis or hemorrhage. Several scattered microcysts contained colloid-like, amorphous eosinophilic material. (**B**) The tumor was covered with an unremarkable surface epithelium. (**C**) Tumor cells merged with normal respiratory epithelium. (**D**) The papillary component showed complex, tightly packed papillary tufts. Slit-like spaces between papillae were even throughout the papillary component. (**E**) Some fibrovascular cores were hyalinized (left half). (**F**) Epithelial cells covering the papillae exhibited nuclear abnormalities resembling those of papillary thyroid carcinoma, such as nuclear membrane irregularity (red circles), intranuclear pseudoinclusions (green circles), and a ground-glass appearance. (**G**,**H**) High-power magnification view of the nuclear morphology; green circles indicate those resembling papillary thyroid carcinoma. Hematoxylin and eosin stain. Original magnification: A, 12.5×; B, 60×; C, 100×; D, 40×; E and F, 200×; G and H, 400×.

Macroscopically, the tumor was soft and exophytic with a focal papillary appearance. No visible necrosis, hemorrhage, or degenerative change was identified. Histologically, the tumor was solid and cystic on the scanning view (Figure 2A) and was partially covered with an unremarkable surface epithelium (Figure 2B). A transition from normal respiratory epithelium to the sheets of tumor cells was identified (Figure 2C). The underlying stroma was infiltrated by the tumor with papillary and glandular growth patterns. The papillary component, comprising approximately one-third of the entire specimen, showed complex, tightly packed papillary tufts with fibrovascular cores (Figure 2D). Some of the cores showed hyaline fibrosis (Figure 2E) and were lined by cuboidal-to-columnar cells showing focal pseudostratification. The tumor cell nuclei were round-to-oval and vesicular and displayed mild-to-focally moderate pleomorphism. Although some of them exhibited prominent nucleoli, most of the tumor cells had single, tiny, and inconspicuous nucleoli. The cytoplasm was pale-to-mildly eosinophilic. Nuclear features resembling those of PTC were observed frequently, including irregular thickened nuclear membranes, nuclear grooves (Figure 2F), intranuclear cytoplasmic pseudoinclusions (Figure 2G,H), and chromatin clearing (ground-glass appearance). These histological features were compatible with those of TLLG-NPPA. Atypical mitosis, coagulative tumor cell necrosis, infarction, lymphovascular invasion, or perineural invasion was not identified.

**Figure 3:**
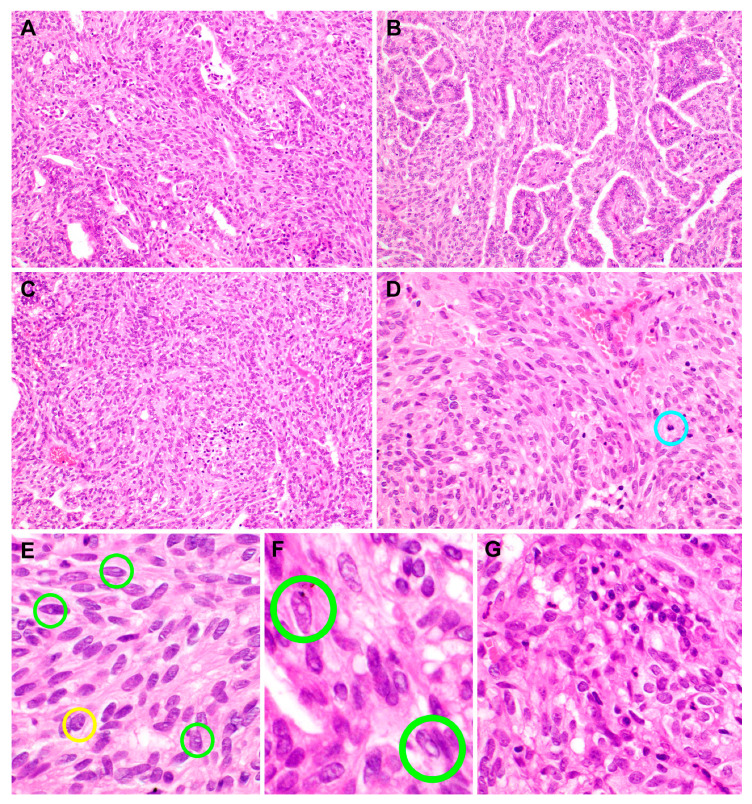
Histopathological features of the spindle cell component of the biphasic thyroid-like low-grade nasopharyngeal papillary adenocarcinoma. (**A**) Anastomosing nests and sheets of tumor cells with spindle-shaped nuclei were intermingled with the papillary component. (**B**) The papillary and spindle cell components merged imperceptibly. (**C**) In some areas, a transition from cuboidal-to-columnar tumor cells to spindle cells was observed. (**D**) Spindle cells were arranged predominantly in a stratified and whorling pattern. The blue circle indicates a mitotic figure. (**E**) Spindle cell nuclei were more elongated and hyperchromatic than the nuclei of epithelial cells covering the papillae. Small intranuclear pseudoinclusions (green circles), prominent nucleoli touching the nuclear membrane (yellow circle), and irregular nuclear membranes were observed. (**F**) High-power magnification view of the nuclear morphology; green circles indicate those resembling papillary thyroid carcinoma. (**G**) Some foci showed intratumoral inflammatory infiltrates. Hematoxylin and eosin stain. Original magnification: A to D, 100×; E,2060×; F, 400×; G, 200×.

In addition to the above-mentioned histological findings, solid anastomosing nests and sheets of tumor cells were intermingled with the papillae (Figure 3A). The papillary and spindle cell components merged imperceptibly throughout the tumor (Figure 3B). In some areas, a transition from cuboidal-to-columnar tumor cells to spindle cells was observed (Figure 3C). The spindle cell component was present both internally and peripherally and was arranged predominantly in a stratified and whorling pattern (Figure 3D). The spindle cell component comprised about two-thirds of the entire tumor volume. The nuclear morphology of the spindle cells was similar to that of the epithelial cells covering the papillae. The spindle cell nuclei were slightly more elongated and hyperchromatic. Nuclear grooves, intranuclear pseudoinclusions (Figure 3E,F), and prominent nucleoli were also observed in the spindle cell component, but less frequently than in the papillary component. Some foci showed intratumoral lymphoplasmacytic infiltrates (Figure 3G).

### 2.3. Immunostaining Results

Immunostaining was performed using the compact polymer method (Bond Polymer Refine Detection kit; Leica Biosystems, Newcastle, UK). Four-micrometer formalin-fixed, paraffin-embedded sections were incubated with primary antibodies against thyroid transcription factor-1, vimentin, Hector Battifora mesothelial-1, cytokeratin 7, cytokeratin 19, paired box 8, p63, smooth muscle actin, S-100, cytokeratin 5/6, thyroglobulin, and BRAF V600E. In situ hybridization for Epstein–Barr virus-encoded small RNAs was also performed.

**Figure 4:**
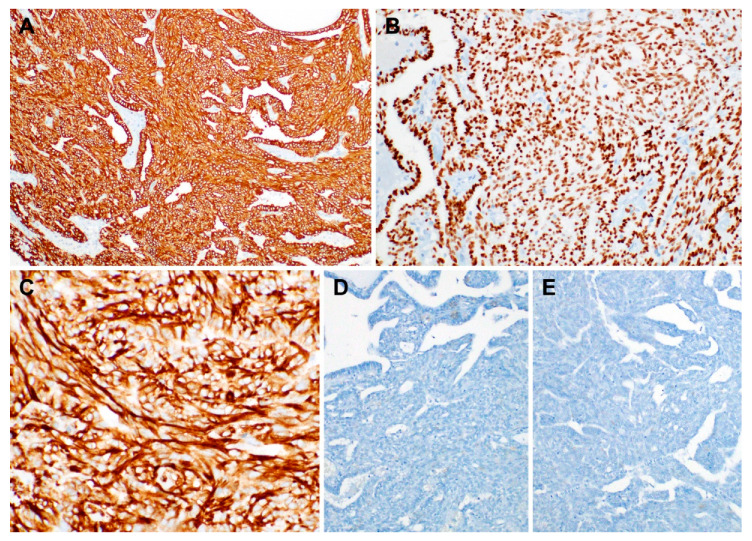
Immunophenotype of biphasic thyroid-like low-grade nasopharyngeal papillary adenocarcinoma. Both papillary and stromal cell components tested uniformly and strongly positive for (**A**) cytokeratin 7, (**B**) thyroid transcription factor-1, and (**C**) vimentin. In contrast, the tumor cells did not express (**D**) thyroglobulin and (**E**) BRAF V600E. Polymer method. Original magnification: A and B, 100×; C, 200×; D and E, 100×.

The results of immunostaining are summarized in Table 1. The tumor cells of both papillary and spindle cell components were diffusely and strongly positive for cytokeratin 7 (Figure 4A), cytokeratin 19, thyroid transcription factor-1 (Figure 4B), vimentin (Figure 4C), and Hector Battifora mesothelial-1. In contrast, the tumor cells were negative for paired box 8, p63, smooth muscle actin, S-100, cytokeratin 5/6, thyroglobulin (Figure 4D), and BRAF V600E (Figure 4E). In situ hybridization for Epstein–Barr virus-encoded small RNAs yielded negative results in the tumor cells of both components. The papillary and spindle cell components showed an identical immunophenotype.

## 3. Discussion

The presence of spindle cells in TLLG-NPPA is an unusual phenomenon. Only five case reports describing six patients with TLLG-NPPA exhibiting spindle cells have been published to date (Table 2) [2,3,5,8,9,10]. The spindle cell component was prominent in only two of those cases [2,8]. Histologically, the spindle cell component did not exhibit severe nuclear pleomorphism or atypical mitoses. In our case, the spindle cell component occupied two-thirds of the entire tumor volume. Due to the small number of cases, it remains unknown whether TLLG-NPPA with prominent spindle cells differs pathogenetically or prognostically from TLLG-NPPA without any spindle cell component. Yokoi et al. reported one of the two cases in which tumors possessed prominent spindle cells. The patient was disease-free at 34 months after the surgery [8]. The follow-up information of another case reported by Petersson et al. [2] is currently unavailable.

Oide et al. [5] recently documented a case of TLLG-NPPA with squamous differentiation. The scattered squamous cell foci showed no obvious nuclear atypia or proliferative activity. They were morphologically similar to the squamous metaplasia observed in PTC, and tested positive for p63, cytokeratin 5/6, and thyroid transcription factor-1. In contrast to their case, the prominent spindle cell components of our case, as well as those reported by Petersson et al. [2] and Yokoi et al. [8] did not express p40 and cytokeratin 5/6, indicating that the development of spindle cells occurred independently of squamous differentiation.

The mechanism underlying abnormal expression of thyroid transcription factor-1 in TLLG-NPPA remains unclear. Thyroid transcription factor-1 is a homeodomain-containing tissue-specific transcription factor that belongs to the *NKX2-1* gene family and is known to play a critical role in cell differentiation and morphogenesis of the lungs and thyroid [11]. Therefore, the specific expression of thyroid transcription factor-1 is commonly used in the diagnosis of primary lung and thyroid carcinomas. However, it has been reported recently that positive thyroid transcription factor-1 immunoreactivity is also observed in carcinomas of the breast, colon, ovary, endometrium, and urinary bladder [12,13]. Further investigations are necessary to explain the abnormal expression of thyroid transcription factor-1.

The diagnostic differentiation between TLLG-NPPA and metastatic PTC is difficult due to their similar morphology. The tumor cells of both lesions have papillary architectures with invasive growth patterns and nuclear atypia. They also exhibit a ground-glass appearance and overlapping nuclei with a clear cytoplasm, nuclear grooves, and psammoma bodies. Immunohistochemically, both tumors yield positive results for cytokeratin 7, cytokeratin 19, vimentin, thyroid transcription factor-1, and Hector Battifora mesothelial-1 [2,3,5,7,8,10,14,15,16]. The transition from the surface epithelium to tumor cell nests and the lack of thyroglobulin and paired box 8 immunoreactivity are the most important findings that distinguish TLLG-NPPA from metastatic PTC. It is well known that thyroglobulin is specific to the thyroid and is expressed only by non-neoplastic and neoplastic thyroid tissue. In addition, almost all cases of thyroid carcinoma are positive for paired box 8, whereas in all the previously reported TLLG-NPPA cases examined for PAX8 immunoreactivity the tumor cells were negative for PAX8 [11]. In our case, ultrasonography of the patient’s thyroid gland did not reveal any abnormality, making the diagnosis of metastatic PTC even less likely. Another important differential diagnosis is polymorphous low-grade adenocarcinoma. Although this tumor has been known to exhibit an indolent clinical behavior, previous studies reported a significant association between the extent of papillary growth in polymorphous low-grade adenocarcinoma and cervical lymph node metastasis [17]. Negative or focally positive S-100 immunoreactivity distinguishes TLLG-NPPA from polymorphous low-grade adenocarcinoma, in which the expression of S-100 is uniform and there is a more than 90% positivity for tumor cells [16].

Although a majority of malignant nasopharyngeal neoplasms are closely associated with Epstein–Barr virus infection, in situ hybridization for Epstein–Barr virus-encoded small RNAs yielded negative results in our case and previously reported cases [11]. Over the last decade, several studies have reported that oncogenic human papillomavirus is associated with a subgroup of nasopharyngeal carcinoma. High- and low-risk human papillomavirus tests were performed in previously reported cases, and yield negative results [11,18]. Due to the limited number of cases, the relationship between TLLG-NPPA and Epstein–Barr virus or human papillomavirus could not be established.

Since TLLG-NPPA exhibits morphological and immunophenotypical features similar to PTC [10], it is reasonable to speculate that TLLG-NPPA has a *BRAF* V600E mutation, the most common mutation observed among patients with PTC. However, there is little information about the genetic features of TLLG-NPPA. Petersson et al. [2] investigated *BRAF* and *KIT* mutational statuses and *SSY*-*SSX1/2* rearrangement in their cases but did not identify any particular genetic alteration. Similarly, we performed BRAF V600E immunohistochemistry which yielded negative results regarding its expression.

The treatment and prognosis of nasopharyngeal adenocarcinoma depend on the tumor type and clinical stage [16,19,20]. The prognosis of TLLG-NPPA as a low-grade malignancy is excellent [4,19]. Since this patient was recently diagnosed and treated, no meaningful follow-up information could be obtained. However, thus far, no case of TLLG-NPPA has been associated with lymphovascular spread or metastasis with a follow-up period of as long as 15 years [2]. Local recurrence may occur with inadequate surgical resection.

## 4. Conclusions

We have presented a case of TLLG-NPPA with a prominent spindle cell component. Both the papillary and spindle cell components displayed similar nuclear features and identical immunophenotypes. Careful histological examination with immunostaining is necessary to make an accurate diagnosis. Pathologists should be aware of this rare lesion to distinguish it from salivary-type adenocarcinomas of the nasopharynx and metastatic PTC, particularly in small biopsy specimens.

## Figures and Tables

**Table 1:** Immunostaining results in the papillary and spindle cell components of biphasic thyroid-like low-grade nasopharyngeal papillary adenocarcinoma.

Antibody	Immunostaining Result	
	Papillary Component	Spindle Cell Component
Thyroid transcription factor-1	Positive	Positive
Vimentin	Positive	Positive
Hector Battifora mesothelial-1	Positive	Positive
Cytokeratin 7	Positive	Positive
Cytokeratin 19	Positive	Positive
Paired box 8	Negative	Negative
p63	Negative	Negative
Smooth muscle actin	Negative	Negative
S-100	Negative	Negative
Cytokeratin 5/6	Negative	Negative
Thyroglobulin	Negative	Negative
BRAF V600E	Negative	Negative
Epstein–Barr virus-encoded small RNAs	Negative	Negative

**Table 2:** Reported cases of biphasic thyroid-like low-grade nasopharyngeal papillary adenocarcinoma with a spindle cell component.

No.	Reference	Age (Years)	Sex	Quantity of Spindle Cell Component	Current Status	Follow-Up (Months)
1	Petersson et al. [2]	39	F	Prominent	NA	Recent
2	Ohe et al. [3]	25	M	NA	NED	13
3	Ohe et al. [3]	41	M	NA	NED	9
4	Oishi et al. [10]	47	F	Focal	NED	19
5	Yokoi et al. [8]	58	M	Prominent	NED	34
6	Mirza et al. [9]	54	M	Several foci of solid aggregates	NED	12
7	The present report	50	F	Prominent	NA	Recent

F, female; M, male; NA, not applicable; NED, no evidence of disease.

## Abbreviations

TLLG-NPPA	Thyroid-like low-grade nasopharyngeal papillary adenocarcinoma
PTC	Papillary thyroid carcinoma

## References

[1] El-Naggar A.K., Chan J.K.C., Grandis J.R., Takata T., Slootweg P.J. (2017). World Health Organization Classification of Head and Neck Tumours.

[2] Petersson F., Pang B., Loke D., Hao L., Yan B. (2011). Biphasic low-grade nasopharyngeal papillary adenocarcinoma with a prominent spindle cell component: Report of a case localized to the posterior nasal septum. Head Neck Pathol..

[3] Ohe C., Sakaida N., Tadokoro C., Fukui H., Asako M., Tomoda K., Uemura Y. (2010). Thyroid-like low-grade nasopharyngeal papillary adenocarcinoma: Report of two cases. Pathol. Int..

[4] Sillings C.N., Weathers D.R., Delgaudio J.M. (2010). Thyroid-like papillary adenocarcinoma of the nasopharynx: A case report in a 19-year-old male. Oral Surg. Oral Med. Oral Pathol. Oral Radiol. Endod..

[5] Oide T., Kadosono O., Matsushima J., Wu D., Nagashima H., Saigusa H., Masunaga A., Nakatani Y., Hiroshima K. (2017). Thyroid-like low-grade nasopharyngeal papillary adenocarcinoma with squamous differentiation: A novel histological finding. Hum. Pathol..

[6] Wenig B.M., Hyams V.J., Heffner D.K. (1988). Nasopharyngeal papillary adenocarcinoma. A clinicopathologic study of a low-grade carcinoma. Am. J. Surg. Pathol..

[7] Carrizo F., Luna M.A. (2005). Thyroid transcription factor-1 expression in thyroid-like nasopharyngeal papillary adenocarcinoma: Report of 2 cases. Ann. Diagn. Pathol..

[8] Yokoi H., Terado Y., Fujiwara M., Matsumoto Y., Ikeda T., Saito K. (2018). Biphasic low-grade nasopharyngeal papillary adenocarcinoma: A case report and literature review. BMC Clin. Pathol..

[9] Mirza R., Dela Cruz N., Herrera G.A. (2020). Thyroid-Like Low-Grade Nasopharyngeal Papillary Adenocarcinoma with Biphasic Histology. Case Rep. Pathol..

[10] Oishi N., Kondo T., Nakazawa T., Mochizuki K., Kasai K., Inoue T., Yamamoto T., Watanabe H., Hatsushika K., Masuyama K. (2014). Thyroid-like low-grade nasopharyngeal papillary adenocarcinoma: Case report and literature review. Pathol. Res. Pract..

[11] Zhang W.L., Ma S., Havrilla L., Cai L., Yu C.Q., Shen S., Xu H.T., Wang L., Yu J.H., Lin X.Y. (2017). Primary thyroid-like low-grade nasopharyngeal papillary adenocarcinoma: A case report and literature review. Medicine.

[12] Jones T.D., Kernek K.M., Yang X.J., Lopez-Beltran A., MacLennan G.T., Eble J.N., Lin H., Pan C.X., Tretiakova M., Baldridge L.A. (2005). Thyroid transcription factor 1 expression in small cell carcinoma of the urinary bladder: An immunohistochemical profile of 44 cases. Hum. Pathol..

[13] Comperat E., Zhang F., Perrotin C., Molina T., Magdeleinat P., Marmey B., Regnard J.F., Audouin J., Camilleri-Broet S. (2005). Variable sensitivity and specificity of TTF-1 antibodies in lung metastatic adenocarcinoma of colorectal origin. Mod. Pathol..

[14] Kakkar A., Sakthivel P., Mahajan S., Thakar A. (2019). Nasopharyngeal Papillary Adenocarcinoma as a Second Head and Neck Malignancy. Head Neck Pathol..

[15] Li M., Wei J., Yao X., Wang C. (2017). Clinicopathological Features of Low-Grade Thyroid-like Nasopharyngeal Papillary Adenocarcinoma. Cancer Res. Treat..

[16] Pineda-Daboin K., Neto A., Ochoa-Perez V., Luna M.A. (2006). Nasopharyngeal adenocarcinomas: A clinicopathologic study of 44 cases including immunohistochemical features of 18 papillary phenotypes. Ann. Diagn. Pathol..

[17] Evans H.L., Luna M.A. (2000). Polymorphous low-grade adenocarcinoma: A study of 40 cases with long-term follow up and an evaluation of the importance of papillary areas. Am. J. Surg. Pathol..

[18] Wu P.Y., Huang C.C., Chen H.K., Chien C.Y. (2007). Adult thyroid-like low-grade nasopharyngeal papillary adenocarcinoma with thyroid transcription factor-1 expression. Otolaryngol. Head Neck Surg..

[19] Ozer S., Kayahan B., Cabbarzade C., Bugdayci M., Kosemehmetoglu K., Yucel O.T. (2013). Thyroid-like papillary adenocarcinoma of the nasopharynx with focal thyroglobulin expression. Pathology.

[20] Fu C.H., Chang K.P., Ueng S.H., Wu C.C., Hao S.P. (2008). Primary thyroid-like papillary adenocarcinoma of the nasopharynx. Auris Nasus Larynx.

